# Potential of Coupling Metaheuristics-Optimized-XGBoost and SHAP in Revealing PAHs Environmental Fate

**DOI:** 10.3390/toxics11040394

**Published:** 2023-04-21

**Authors:** Gordana Jovanovic, Mirjana Perisic, Nebojsa Bacanin, Miodrag Zivkovic, Svetlana Stanisic, Ivana Strumberger, Filip Alimpic, Andreja Stojic

**Affiliations:** 1 Institute of Physics Belgrade, National Institute of the Republic of Serbia, University of Belgrade, 11000 Belgrade, Serbia; mirjana.perisic@ipb.ac.rs (M.P.); filip.alimpic@ipb.ac.rs (F.A.); andreja.stojic@ipb.ac.rs (A.S.); 2 Faculty of Informatics and Computing, Singidunum University, 11000 Belgrade, Serbia; nbacanin@singidunum.ac.rs (N.B.); mzivkovic@singidunum.ac.rs (M.Z.); istrumberger@singidunum.ac.rs (I.S.)

**Keywords:** machine learning, extreme gradient boosting, metaheuristics optimization, swarm intelligence, explainable artificial intelligence, sine cosine algorithm, benzo(a)pyrene

## Abstract

Polycyclic aromatic hydrocarbons (PAHs) refer to a group of several hundred compounds, among which 16 are identified as priority pollutants, due to their adverse health effects, frequency of occurrence, and potential for human exposure. This study is focused on benzo(a)pyrene, being considered an indicator of exposure to a PAH carcinogenic mixture. For this purpose, we have applied the XGBoost model to a two-year database of pollutant concentrations and meteorological parameters, with the aim to identify the factors which were mostly associated with the observed benzo(a)pyrene concentrations and to describe types of environments that supported the interactions between benzo(a)pyrene and other polluting species. The pollutant data were collected at the energy industry center in Serbia, in the vicinity of coal mining areas and power stations, where the observed benzo(a)pyrene maximum concentration for a study period reached 43.7 $\mathrm{ng}\;m^{-3}$. The metaheuristics algorithm has been used to optimize the XGBoost hyperparameters, and the results have been compared to the results of XGBoost models tuned by eight other cutting-edge metaheuristics algorithms. The best-produced model was later on interpreted by applying Shapley Additive exPlanations (SHAP). As indicated by mean absolute SHAP values, the temperature at the surface, arsenic, PM${}_{10}$, and total nitrogen oxide (NOx) concentrations appear to be the major factors affecting benzo(a)pyrene concentrations and its environmental fate.

## 1. Introduction

Polycyclic aromatic hydrocarbons (PAHs) refer to a group of several hundred species with two to seven fused benzene rings, generated via incomplete combustion of organic substances, in the high temperature or pressure process. The majority of these polluting species are persistent, bioaccumulative, light sensitive, heat and corrosion resistant, and emitted from both natural and anthropogenic sources, with the latter being dominant in urban areas.

The concentrations of PAHs in the atmosphere are dependent on the number and quality of air pollutant emission sources, regional meteorological conditions, season, measurement site characteristics, as well as other factors which contribute to their dispersion and have an impact on atmospheric chemistry, dry or wet deposition, and finally, pollutant half-lives and their mutual interactions [1].

After being released, mostly as part of vehicle exhaust and emissions from biomass and fossil fuel burning, PAHs are distributed to all environmental compartments, adsorbed to airborne particle matter, and deposited on terrestrial and water surfaces. Their concentrations are particularly high in the cold season, as a result of increased fossil fuel burning, reduced thermal and photo-decomposition, and a low planetary boundary layer. Apart from their common sources in urban areas, Hoffer et al. [2] reported that municipal incineration of plastic waste in urban areas emits up to 750 more PAHs than the combustion of dry firewood under the same conditions, and estimated that these emissions were dominated by 4–6 ring PAHs, which are up to 4100 times more toxic than the ones emitted from wood combustion.

In the atmosphere, PAHs are found in the gaseous phase or, more often, adsorbed onto suspended particles. The U.S. EPA has listed 16 compounds as the “priority PAHs” due to their adverse health effects, frequency of occurrence, and potential for human exposure. As regards their impacts on human health, PAHs have obtained significant attention due to the toxicity of low molecular weight species, being the most abundant in the gas phase, and the carcinogenic potential of heavy molecular weight compounds, being mostly particle-bound [3]. The smaller the particle size, the higher the share of carcinogenic PAHs, and thus fine aerosol fraction poses excessive risks to human health. In addition to this, PAHs contribute to the high mutagenicity and carcinogenicity of suspended particles through reactions with atmospheric oxidants, which result in the formation of secondary species [4].

In Serbia, the use of low-quality lignite coal is the major cause of low air quality. While domestic fuel burning (wood, coal, and gas) contributes to global PM${}_{2.5}$ and PM${}_{10}$ emissions with 20% and 15%, respectively. Karagulian et al. [5] estimated that these contributions amount to 32% and 45% in Central and Eastern Europe, respectively. Since it has been recognized as an indicator of total exposure to carcinogenic PAHs, the benzo(a)pyrene (B[a]P) presence is regularly monitored. To prevent and reduce harmful effects on human health and the environment, a European Directive has set a target value of 1 $\mathrm{ng}\;m^{-3}$ for the total content of B[a]P in the PM${}_{10}$ fraction, averaged over a calendar year. In this study, we have used the pollutant data from Lazarevac, an energy industry center in the vicinity of the coal mining areas and power stations, where the observed B[a]P concentrations have occasionally reached 30 $\mathrm{ng}\;m^{-3}$. In comparison to this, Elzein et al. [6] reported the ∑17-PAHs concentrations have ranged from 2.6 and 31.2 $\mathrm{ng}\;m^{-3}$, and 8.4 to 42.9 $\mathrm{ng}\;m^{-3}$, in Beijing and Delhi, respectively. Previous studies have confirmed that the residents of the coal mining regions face an incremental lifetime cancer risk which is significantly higher than the target value [7].

In this study, based on our previous research [8,9,10,11,12,13,14,15], we have applied a novel approach based on the XGBoost model to identify the factors which are mostly associated with the observed B[a]P concentrations and the environmental conditions which support and facilitate B[a]P level dynamics and its interactions with other polluting species. The XGBoost itself is an efficient model; nevertheless, its hyperparameters require tuning for each particular prediction task in order to achieve good performance on the observed dataset. Manual tuning of the hyperparameters is an extremely slow, time-consuming, and error-prone task that is considered to be NP-hard by nature. To address this, a variant of the SCA metaheuristics algorithm [16] has been used to optimize the XGBoost hyperparameters. Metaheuristics algorithms, being stochastic by nature, have been established as a common choice to tackle NP-hard challenges. By performing simulations for the sake of this research, the most promising metaheuristics algorithm was determined to be the sine cosine algorithm (SCA); in other words, it was selected empirically. Moreover, this paper also proposes a modified version of SCA, by hybridizing it with another algorithm, to cancel the limitations of the elementary SCA. Modified SCA was later utilized as a part of the machine learning framework, and tasked to tune the collection of the XGBoost hyperparameters for this problem. The results attained by the proposed model have been compared to the results of XGBoost models tuned by eight other cutting-edge metaheuristics algorithms. The best-produced model was later on interpreted by applying Shapley Additive exPlanations (SHAP).

## 2. Background

### 2.1. XGBoost

The XGBoost algorithm utilizes an adaptive training method for optimizing its objective function, where each step in the optimization process relies on the outcome of the previous step. The mathematical representation of the XGBoost model’s objective function has been defined as follows:

(1) $${F_{o}}^{i}=\sum_{k=1}^{n}l\left(y_{k},{{\hat{y}}_{k}}^{i-1}+f_{i}\left(x_{k}\right)\right)+R\left(f_{i}\right)+C,$$

where the *t*-th round loss is denoted by *l*, $y_{k}$ and ${\hat{y}}_{k}$ denote target observed values and predictions, respectively; $f_{i}$ are additive functions from the space of the regression trees, constant term is marked as *C*, while the model’s regularization parameter *R* can be defined as:

(2) $$R\left(f_{i}\right)=\gamma T_{i}+\frac{\lambda }{2}\sum_{j=1}^{T}w_{j}^{2}$$

where *T* corresponds to the number of leaves in the tree, while *w* values denote the scores in the corresponding leaves [17].

In general, the complexity of the tree structure is inversely proportional to the values of the customization parameters γ and λ. The larger the values of these parameters, the simpler the tree structure becomes. The model’s first and second derivatives, represented by *g* and *h*, respectively, are expressed as follows:

(3) $$g_{j}=\partial_{{{\hat{y}}_{k}}^{i-1}}l\left(y_{j},{{\hat{y}}_{k}}^{i-1}\right)$$

(4) $$h_{j}=\partial_{{{\hat{y}}_{k}}^{i-1}}^{2}l\left(y_{j},{{\hat{y}}_{k}}^{i-1}\right)$$

The solution is obtained using the next two formulas:

(5) $$w_{j}^{*}=-\frac{\sum g_{t}}{\sum h_{t}+\lambda }$$

(6) $${F_{o}}^{*}=-\frac{1}{2}\sum_{j=1}^{T}\frac{{\left(\sum g\right)}^{2}}{\sum h+\lambda }+\gamma T,$$

where the loss function score is denoted by ${F_{o}}^{*}$, while the solution’s weight values are marked by $w_{j}^{*}$.

### 2.2. Metaheuristics Optimization

NP-hard challenges, a common occurrence in computer science, necessitate the use of stochastic algorithms like metaheuristics because deterministic methods are impractical. Metaheuristics methods can be categorized into various families with respect to the natural phenomenon they utilize to steer the search process, such as evolution or insect behavior [18,19,20]. The most significant families of metaheuristic algorithms are nature-inspired methods (further divided into genetic algorithms and swarm intelligence), methods established upon certain physical phenomena (e.g., storm, gravity, electromagnetism), algorithms that imitate certain aspects of the human behavior (e.g., teaching and learning, or brainstorming, or actions taken on social media), and approaches based on mathematical laws to guide the search (e.g., trigonometric function oscillations).

Swarm intelligence is established upon the behavior manifested by massive groups comprised of relatively modest units; for example, insects or birds in swarms, that are able to manifest highly coordinated and sophisticated behavioral patterns while they hunt, feed, mate or migrate [21,22]. These algorithms have exhibited high efficiency in solving a variety of the real-world NP-hard challenges. Among many available algorithms, well-known samples are the particle swarm optimization (PSO) [23], the ant colony optimization (ACO) [24], the firefly algorithm (FA) [25] and the bat algorithm (BA) [26,27]. Recently, a highly efficient group of algorithms were derived from the mathematical functions and their properties to steer the search procedure, including the sine-cosine algorithm (SCA) [16] and the arithmetic optimization algorithm (AOA) [28].

The reason why there is a range of population-based algorithms is due to the no-free-lunch theorem (NFL) [29]. The NFL discloses that no single method can be the best for all optimization problems. Consequently, one algorithm may excel in one task but entirely fail in another, leading to the need for diverse metaheuristics approaches and the requirement to choose a suitable method for each specific optimization challenge.

Recently, population-based algorithms have been a common choice to address numerous real-world problems. The application domains include predicting the number of COVID-19 cases [30,31], fog, cloud and cloud-edge computing systems organization [32,33,34,35], wireless sensors and IoT optimization [36,37,38,39], feature selection [40], image processing and classifying in medicine [41,42], global tuning challenges [43,44], credit card fraud identification [45,46], tracking and predicting air pollution [47,48], network and computer systems intrusion detection [49,50], and finally, tuning different ML structures [51,52,53,54,55,56].

### 2.3. Shapley Additive Explanations

To explain the obtained best-performing model, which is vital for understanding the process being modeled, we have applied the explainable artificial intelligence method SHAP. Avoiding the trade-off between accuracy and interpretability, SHAP provides a straightforward and meaningful interpretation of the model-derived decisions. It is based on Shapley values, calculated as a feature importance measure by a game-theory approach which provides an impact of features on individual predictions [57]. Apportioning the difference between the prediction and the average prediction among the features [58], Shapley values represent fairly distributed payouts among the cooperating players (features) depending on their contribution to the joint payout (prediction). SHAP assigns each feature importance as a measure of its contribution to a particular prediction and interprets the impact compared to a model’s prediction if that feature took some baseline value (mean). This way, the method provides valuable insights into a model’s behavior (1) overcoming the main drawback of inconsistency, (2) minimizing the possibility of underestimating the importance of a feature with a specific attribution value, and (3) capturing feature interaction effects based on a generalization of Shapley values and interpreting the model’s global behavior while retaining local faithfulness [15,59]. The main challenges of the method include Shapley value computation and background data choice which can induce uncertain or unintuitive feature attributions.

In this study, we have used the relative SHAP values introduced by Stojic et al. [11] to gain an insight into relative relationships among feature attributions for each prediction. Relative SHAP values, defined as a share of absolute SHAP in total attributed importance of all features for the particular prediction, show the relative influence of a feature on the prediction.

We have used the Python SHAP implementation (SHAP Python package) and TreeExplainer [59] to obtain SHAP values that we have used to produce SHAP dependency plots, representing the change of feature importance over its value range.

## 3. Methods

### 3.1. Measurements Methods

For this study, the two-year daily concentrations (2018–2019; 645 observations) of particulate matter PM${}_{10}$, its constituents (Pb, As, Cd, Ni, and B[a]P), and inorganic gaseous pollutants (NO, NO${}_{2}$, NOx, and SO${}_{2}$) were obtained from the regulatory air quality monitoring station (44${}^{{}^{\circ}}$23′02″ N, 20${}^{{}^{\circ}}$15′55″ E) in Lazarevac (Serbia). The meteorological data were obtained from the Global Data Assimilation System (GDAS1) with a 1.0${}^{{}^{\circ}}$× 1.0${}^{{}^{\circ}}$ spatial resolution for the longitude and latitude of the monitoring station.

The Sven Leckel SEQ 47/50-RV sampler was used for collecting 24-h samples of particulate matter. The mass concentrations of PM${}_{10}$, Pb, As, Cd, Ni, and B[a]P were determined according to the standards EN 12341, EN 14902, and EN 15549, while the concentrations of NO, NO${}_{2}$, NOx, and SO${}_{2}$ were obtained in accordance with the sampling procedures standardized in EN 14211 and EN 14212.

PM${}_{10}$ were collected on quartz filters (Whatman QMA, 47 mm) daily, as described in the Standard SRPS EN 12341:2015 (Ambient air—Standard gravimetric measurement method for the determination of the PM${}_{10}$ or PM${}_{2.5}$ mass concentration of suspended particulate matter, 2015). The filters were pre-fired to remove organic impurities, and the pre-conditioning of both non-exposed and loaded filters was performed prior to gravimetric measurements.

The concentrations of As, Cd, Cr, Ni, and Pb as PM${}_{10}$ constituents were determined as described in the EN 14902:2008/AC:2013 Standard (Ambient air quality—Standard method for the measurement of Pb, Cd, As, and Ni in the PM fraction of suspended particulate matter, 2008). Firstly, the CEN/TC 264 N779 procedure was applied for the extraction of the trace elements. In brief, the pieces of exposed quartz filters were treated with an acidic mixture of HNO${}_{3}$(c)/30% H${}_{2}$O${}_{2}$/H${}_{2}$O (3/2/5) using analytical grade reagents (Merck) and distilled/deionized water (MiliQ, 18.2 MΩ). The filters were digested in closed 100 mL Teflon vessels in the Anton Paar 3000 microwave accelerated reaction system and the concentrations of trace elements were determined by inductively coupled plasma–mass spectrometry (ICP-MS) (device Agilent 7500ce with Octopole Reaction System). Quality control and verification of the applied procedures for microwave digestion and multi-elemental trace analysis using ICP-MS was conducted using the 2783 NIST (National Institute of Standard and Technology, MD, USA) standard reference material analysis, containing a PM${}_{10}$ fraction of urban dust from a mixed industrial urban area of Vienna, collected on a polycarbonate membrane filter. The recovery values were within the satisfactory range of ±20% from the reference value.

B[a]P was determined by the procedure described in the SRPS ISO 12884:2010 Standard (Ambient air—Determination of total (gas and particle-phase) polycyclic aromatic hydrocarbons—Collection on sorbent-backed filters with gas chromatographic/mass spectrometric analyses, 2010). Parts of the exposed filters underwent a microwave extraction procedure with a solvent mixture of n-hexane and acetone (12.5 mL n-hexane: 12.5 mL acetone) according to EPA method 3546. After extraction, the solution volume was reduced by rotary evaporation under reduced pressure (55.6 kPa and 0.2 mL iso-octane) to 1 mL. Afterward, the n-hexane solution was reduced to 0.25 mL under a nitrogen stream. Known quantities of internal standards were added to estimate the method recovery. B[a]P was analyzed using gas chromatography coupled with a mass selective detector (Agilent GC 6890/5973 MSD) according to the EPA compendium method TO-13A with a DB-5 MS capillary column (30 m × 0.25 mm × 25 μm). The oven temperature program started at 70 ${}^{{}^{\circ}}$C (duration of 4 min) and ramped 8 ${}^{{}^{\circ}}$C min-1 to the end temperature of 310 ${}^{{}^{\circ}}$C (duration of 5 min). The solvent delay was 5 min and the run time was 46 min. The calibration curve was obtained by spiking seven different quantities of B[a]P, all with an R2 of the calibration curve above 0.995. Recovery values ranged from 85% to 110% for all the PAHs contained in the internal standard.

The samples were collected at the suburban site located in the energy industry center of Lazarevac, a municipality of Belgrade (Serbia), and a home to 60,000 residents. The sampling location, surrounded by residential areas and sports facilities, is exposed to mining pollutant sources and emissions from household coal and wood fireboxes. Additionally, the nearest coal mine Vreoci and regional power station are located around 5 km east and northeast, while the 80 square kilometers large coal mining area Kolubara, which supplies 75% of Serbia’s electricity generation and largest state power plants Nikola Tesla A and B, are located around 10 km north and 30 km northwest of the sampling site, respectively.

### 3.2. Original Sine Cosine Algorithm

The algorithm, proposed by Mirjalili in 2016 [16], is based on the properties of elementary trigonometrical functions. SCA belongs to the group of population-based metaheuristics that starts each run by producing a set of arbitrary initial solutions within the scope of the search realm. The individual positions update following the swinging behavior of the sine and cosine functions over time. SCA conducts the exploration and exploitation mechanisms steered by the set of four arbitrary control parameters. The fundamental SCA search is mathematically defined by Equation (7):

(7) $$X_{i}^{t+1}=\left\{\begin{array}{c} X_{i}^{t+1}=X_{i}^{t}+r_{1}\cdot sin\left(r_{2}\right)\cdot \left|r_{3}\cdot P_{i}^{*t}-X_{i}^{t}\right|,\;r_{4}<0.5\\ X_{i}^{t+1}=X_{i}^{t}+r_{1}\cdot cos\left(r_{2}\right)\cdot \left|r_{3}\cdot P_{i}^{*t}-X_{i}^{t}\right|,\;r_{4}\geq 0.5, \end{array}\right.$$

where $X_{i}^{t}$ and $X_{i}^{t+1}$ define the individual’s position in *i*-th dimension in a pair of consecutive iterations *t* and i+1, respectively, $r_{1-4}$ are four generated above-mentioned control parameters, the $P_{i}^{*}$ defines the target’s position (the most recent estimation of the optimal solution) within *i*-th dimension. Additionally, the fresh values for $r_{1-4}$ parameters are summoned for each component of each solution within the population.

### 3.3. Proposed Modified Sine Cosine Algorithm

The core implementation of SCA is considered to be an exceptional optimizer; however, like other metaheuristics methods, it also has some drawbacks. Testing using benchmark sets has shown that SCA is effective at exploring solutions, but lacks the ability to effectively exploit these solutions in the later stages of the process. This results in a limited exploration when the algorithm should be focusing on the most promising areas. In contrast, the firefly algorithm (FA) is known for its superior exploitation capability, as described by [25].

This manuscript suggests a hybrid solution by combining SCA and FA algorithms, aiming to profit from the advantages of both metaheuristics, aiming to cancel out each other’s disadvantages. At the start of the execution, the solutions within the population will update according to the SCA search procedure, as described by Equation (7). However, in later stages, when it is necessary to narrow down and exploit the favorable regions of the search realm, the exploitation phase is backed up by employing the powerful FA search mechanism, defined by Equation (8):

(8) $$X_{i}^{t+1}=X_{i}^{t}+\beta_{0}\cdot e^{-\gamma r_{i,j}^{2}}\left(X_{j}^{t}-X_{i}^{t}\right)+\alpha^{t}\left(\kappa -0.5\right)$$

where α represents the randomization variable, κ is an arbitrary value drawn from the Gaussian distribution. Finally, the space between solutions *i* and *j* is denoted as $r_{i,j}$.

A couple of new control parameters have been suggested to steer the alternation between the two search procedures in the later stage of the execution. The varying search vs control parameter is used to activate the combined search mode in the case where t>vs, when the suggested approach should alternate between SCA and FA search methods. Variable vs is initially set as maxIter/5, that has been determined empirically.

The second control parameter, named search mode sm, determines for every individual solution in the population whether to proceed with the SCA or FA search option. Each solution produces a random value rnd in range [0,1], and if rnd<sm it will perform an SCA search, or otherwise continue with the FA search option. The value of this parameter is dynamically reduced over the iterations, giving an additional focus on a stronger FA search in the latter rounds. Initially, sm is set to 0.8, being reduced over time according to Equation (9).

(9) $$sm_{t}=sm_{t-1}-\left(sm_{t-1}/10\right)$$

The hybrid algorithm is labeled hybrid self-adaptive SCA (HSA-SCA), and its pseudocode summarizing the most significant steps of the approach is provided by Algorithm 1.
**Algorithm 1** Pseudocode of the HSA-SCA metaheuristics**Spawn** a collection of starting solutions (X)**while** t<maxIter **do**   **validate** each individual in terms of its fitness   **for** each individual inside (X) **do**     **if** t<vs **then**        Perform SCA search mechanism, provided by Equation (7)     **else**        **if** rnd<sm **then**          Perform SCA search mechanism, provided by Equation (7)        **else**          Perform FA search mechanism, provided by Equation (8)        **end if**     **end if**   **end for****end while****return** The current fittest solution determined as the global optimum

## 4. Experimental Findings and Comparative Analysis

This section first provides insights into the dataset preprocessing, implementation technology, and evaluation metrics used to evaluate different tuned XGBoost models, followed by experimental setup, results, and comparative analysis. Finally, to validate improvements of devised hybrid metaheuristics over other baseline cutting-edge approaches, statistical tests were conducted, as suggested in the state-of-the-art AI literature [60].

### 4.1. Dataset Preprocessing, Implementation Technology and Evaluation Metrics

As already pointed out in Section 3, the employed dataset includes 645 observations. The challenge is formulated as a regression problem, where the feature with daily values for B[a]P was set as the target. Since the XGBoost is a tree-based method, scaling values, e.g., within the range [0,1], were not needed; therefore original measured values were used.

**Figure 1:**
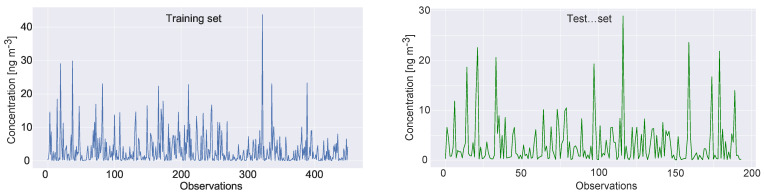
The Benzo(a)pyrene feature dataset split.

However, since the XGBoost requires training, the dataset was divided into train and test, where 70% of observations were used for training and 30% for testing. The same split was used for all metaheuristics considered for comparative analysis and the same pseudo-random number seed was employed, with the goal of establishing fair comparison conditions. It is noted that during the pre-experimentation, simulations with validation test were also conducted; however, improvements could not be achieved, and therefore it was decided to proceed with only training and testing data. Visual representation of the dataset split for the target variable is shown in Figure 1.

The analysis was conducted using daily concentrations and daily mean meteorological parameters, which have minimal to no autocorrelation in such a short period. Moreover, atmospheric processes relevant to air pollution dynamics usually occur within an hour, making autocorrelations even less prominent when using daily data.

The simulation environment, along with all methods, was implemented in Python using data science and ML libraries: *numpy*, *pandas*, *scikitlearn*, *xgboost*, *matplotlib*, *seaborn* and *shap*. Code snippets of the simulation framework along with the best generated XGBoost model by proposed HSA-SCA approach is available at the following URL: https://doi.org/10.5281/zenodo.7831739 (accessed on 25 February 2023).

The XGBoost model’s experimental results have been evaluated by a set of traditional machine learning metrics, including mean squared error (MSE) defined by Equation (10), root mean squared error (RMSE) obtainable by Equation (11), mean absolute error (MAE) calculated by Equation (13), and the coefficient of determination (R2) described with Equation (13).

(10) $$MSE=\frac{1}{N}\sum_{i=1}^{N}{\left(\hat{a_{i}}-a_{i}\right)}^{2}$$

(11) $$RMSE=\sqrt{\frac{1}{N}\sum_{i=1}^{N}{\left(\hat{a_{i}}-a_{i}\right)}^{2}}$$

(12) $$MAE=\frac{1}{N}\sum_{i=1}^{N}\left|\hat{a_{i}}-a_{i}\right|$$

(13) $$R2\;=1-\;\frac{\sum_{i\;=1}^{n}\;{\left(a_{i}-\hat{a_{i}}\right)}^{2}}{\sum_{i\;=1}^{n}\;{\left(a_{i}-\bar{a}\right)}^{2}},$$

where $a_{i}$ and $\hat{a_{i}}$ represent arrays comprised of the observed values that are predicted, and predicted values, both with length *N*. This paper utilizes MSE as the fitness function that is required to be minimized.

Additionally, according to [61,62], the index of agreement (IA) can be an insightful statistical measure used to evaluate the performance of a model or forecast in predicting a particular event or phenomenon, as well as the metric for the best-generated models. The IA can be calculated as the ratio of the MSE and the potential error that is varying in range [0,1], where the value of 1 suggests perfect agreement, while the value of 0 suggests no match at all. The Equation (14) shows how the IA value is obtained:

(14) $$IA\;=1-\;\frac{\sum_{i\;=1}^{n}\;{\left(a_{i}-\hat{a_{i}}\right)}^{2}}{\sum_{i\;=1}^{n}\;{\left(\left|\hat{a_{i}}-\bar{a}\right|+\left|a_{i}-\bar{a}\right|\right)}^{2}},0\leq IA\leq 1,$$

where $a_{i}$ and $\hat{a_{i}}$ again denote arrays comprised of the observed and predicted values, and the $\bar{a}$ are average observed values.

### 4.2. Experimental Setup

The proposed HSA-SCA algorithm was tasked to optimize the XGBoost model for this particular dataset. The set of optimized XGBoost hyperparameters, accompanied by their corresponding search limits and variable types are provided as follows:

- learning rate (η), search limits: [0.1,0.9], continuous variable,
- min_child_weight, search limits: [1,10], continuous variable,
- subsample, search limits: [0.01,1], continuous variable,
- collsample_bytree, search limits: [0.01,1], continuous variable,
- max_depth, search limits: [3,10], integer variable and
- gamma, search limits: [0,0.8], continuous variable.

The parameter counts of the *softprob objective function* (‘num_class’:self.no_classes)were passed as a parameter to the XGBoost, while the remainder of the XGBoost parameters were set to XGBoost defaults during the simulations.

The suggested method has been implemented in the Python programming language, accompanied by the standard collection of Python libraries related to machine learnin including scipy, numpy, and pandas, while the XGBoost model was acquired from the scikit-learn package.

The proposed setup utilizes the solutions’ encoding scheme that observes each solution as an array with length *l*, where *l* denotes the number of optimized hyperparameters. Hence, the value *l* has been set to six, to match the tuned parameters.

Aiming to validate the performance of the XGBoost model tuned by the suggested HSA-SCA algorithm, the achieved results were compared to the results attained by eight other contending powerful algorithms. The comparisons were executed with elementary SCA, genetic algorithm (GA) [63,64], PSO [23], ABC [65], FA [25], whale optimization algorithm (WOA) [66], harris hawks’ optimization (HHO) [67] and chimp optimization algorithm (ChOA) [68]. Every contending algorithm has been implemented independently by the authors of this manuscript, with the control parameters set to the recommended values from their respective publications. Each algorithm has been given the same task, to tune the same set of XGBoost hyperparameters.

All metaheuristics algorithms were tested with 40 solutions in the population and 20 iterations per run, over the course of 20 separate runs. As previously noted, MSE was set as the fitness function that needs to be minimized.

### 4.3. Experimental Findings and Comparative Analysis

This section yields the attained experimental outcomes, for the observed HSA-SCA algorithm and other contenders. Table 1 and Table 2 show the simulation outcomes with respect to the fitness function, accompanied by the detailed metrics achieved in the best individual run of each algorithm, where the best results in each category are marked in bold.

Table 1 shows detailed comparisons with respect to the fitness function (MSE) attained by XGBoost models optimized by the nine regarded algorithms (the proposed HSA-SCA and eight contenders). The results suggest that the HSA-SCA method displayed a supreme performance level, by achieving the best scores for key performance indicators (best, worst, mean, and median). FA scored the best results for standard deviation and variance, by delivering the most stable results. The second best result with respect to the best, worst, mean and median run values was also FA, followed by PSO and ChOA. The best attained score by the HSA-SCA XGBoost model was the MSE of 2.468293, and $R^{2}$ of 0.892845.

Table 2 presents the detailed metrics achieved in the best single run of all regarded algorithms. Once more, it can clearly be seen that the proposed HSA-SCA dominantly outperformed contenders in terms of all indicators—$R^{2}$, R, MSE, RMSE and IA, except MAE, where FA achieved the best score. Looking into the MSE that has been employed as the fitness function with a goal to minimize it, HSA-SCA exhibited superior performance with the score of 2.468293, in front of the FA that scored 2.590850, PSO in third place that achieved 3.077008, and ChOA finishing fourth with the score of 3.129932. In terms of the IA metric, the proposed HSA-SCA was also superior, attaining the value of 0.970348. FA finished second, with IA value of 0.967438, while WOA was third with IA value of 0.961817.

Lastly, the sets of the XGBoost hyperparameters that have been established by the best run of every algorithm are provided within the Table 3. The best performing method, that was the proposed HSA-SCA, produced the XGBoost model with a learning rate of 0.535844, max_child_weight of 4.768378, a subsample of 0.920331, collsample_bytree of 0.899994, max_depth of 5, and gamma value of 0.037125. The XGBoost structure produced by the FA algorithm, that finished in second place, consisted of the learning rate of 0.473028, max_child_weight of 5.459757, a subsample value of 0.937122, collsample_bytree of 1.000000, max_depth value of 7, and finally, gamma value of 0.318114.

**Figure 2:**
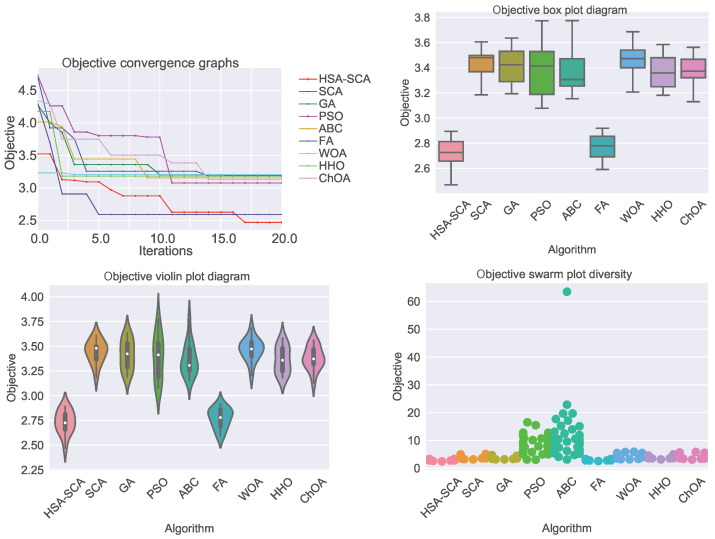
Visualized XGBoost results for all nine metaheuristics in terms of the convergence, box plot, violin diagrams, and swarm diversity plots for the fitness function (MSE).

**Figure 3:**
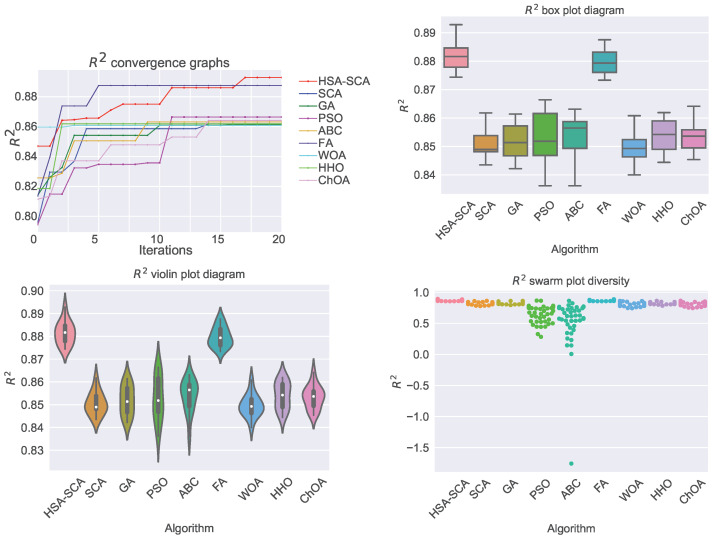
Visualized XGBoost results for all nine metaheuristics in terms of the convergence, box plot, violin diagrams, and swarm diversity plots for the $R^{2}$ indicator.

The performed simulations are visualized in Figure 2 and Figure 3, showing the convergence graphs, box plots, violin plots and swarm plots of all nine algorithms, for both fitness function (Figure 2) and R2 (Figure 3).

While looking into the Figure 2 and Figure 3, it is possible to see that the HSA-SCA method exhibits a very fast converging speed, together with FA metaheuristics, that is a little bit faster at the beginning, but finishing behind HSA-SCA at the end. One can note that FA also exhibits the most stable results, closely followed by the WOA and SCA, as can be seen from the box plot diagrams. Finally, the swarm plots show the diversity of the population within the last round of execution of the best run of each algorithm. It is possible to conclude that all solutions of the HSA-SCA population were proximal to the optimum value.

**Figure 4:**
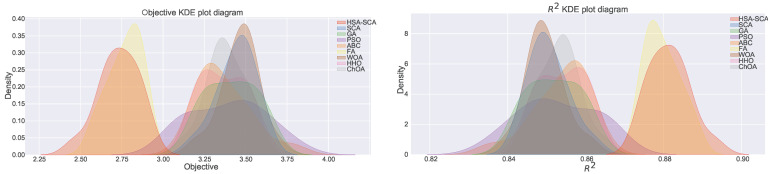
KDE diagrams for MSE (**left**) and $R^{2}$ indicator (**right**).

**Figure 5:**
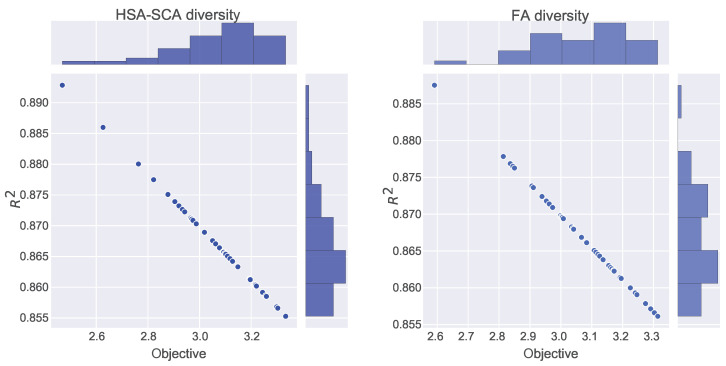
Join plots with histograms of two best methods: HSA-SCA (**left**) and FA (**right**).

Figure 4 depicts the kernel density estimation (KDE), representing the estimation of the probability density function. It can be noted from these plots that the results originate from the normal distribution. Additionally, join plots of both fitness function (MSE) and R2 containing histograms for the two best algorithms (HSA-SCA and FA) are shown in Figure 5.

**Figure 6:**
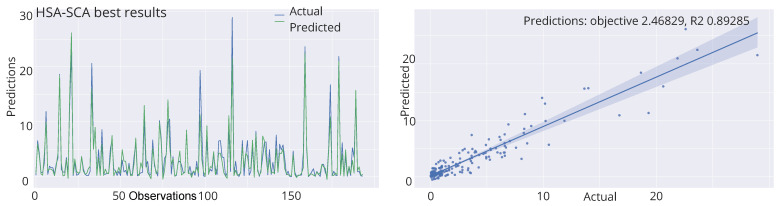

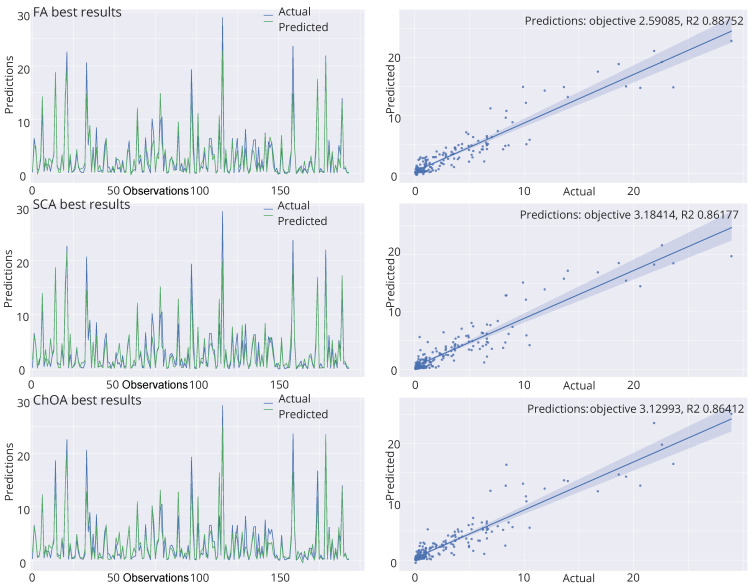
Best-predicted outcomes by the best produced models of HSA-SCA, FA, SCA and ChOA algorithms.

Finally, the visualizations of the best-predicted outcomes attained by the best-produced model by four best algorithms is shown in Figure 6. Once more, it can be concluded that the model optimized by the HSA-SCA algorithm produced the best predictions for the observed problem.

### 4.4. Results Improvements Validation—Statistical Tests

To further evaluate the obtained simulation results and determine whether or not they are statistically significant, the best scores of each of 20 runs from each observed metaheruistics were gathered and inspected as a data series. At the beginning, it was necessary to decide what sort of statistical tests was suitable—parametric or non parametric. First, the safe usage of parametric tests is checked, by evaluation of the independence, normality, and homoscedasticity of the data variances [69]. The independence condition is satisfied, because every run of the metaheuristics algorithms begins by producing a collection of random individuals. Considering the second condition, homoscedasticity, Levene’s test [70] was executed, and since the *p*-value of 0.65 was obtained in every case, it was safe to assume that the homoscedasticity requirement was also fulfilled.

The normality condition has been investigated by employing the Shapiro-Wilk single problem analysis [71]. Shapiro-Wilk *p*-values were independently calculated in terms of each of the observed methods. The established *p*-values for every algorithm were greater than 0.05, therefore it was safe to conclude that it was not possible to reject the H0 hypothesis for both alpha=0.05 and alpha=0.1. As a consequence, it was also possible to conclude that the observed values originated from the normal distribution. One could establish a similar conclusion by simply taking a look at the KDE plots shown in Figure 4. The Shapiro-Wilk test scores are provided within Table 4.

After verifying that the normality requirement was fulfilled, it was safe to conclude that one can proceed by applying the parametric tests. This paper utilizes the paired-*t* test [72], which is frequently selected to evaluate metaheuristics methods [73]. Paired-*t* test can be utilized if it is possible to observe the set of data points as paired measurements, and the differences among the pairs follows a normal distribution. In other words, the variances between samples for every pair of algorithms are required to be normally distributed. To inspect this condition, the Shapiro-Wilk test was employed one more time, over the absolute differences between distributions of the proposed algorithm and other contending methods. The obtained Shapiro-Wilk *p*-values were in every instance greater than the threshold value alpha=0.05, meaning that H0 hypothesis cannot be rejected, and the set of observed values originates from the normal distribution. Since this is the prerequisite for using the paired-*t* test, it is safe to use it and compare the proposed algorithm against each of the opposing methods. The summarized results of both Shapiro-Wilk *p*-values calculated as the prerequisite for the paired-*t* test, and the paired-*t* test itself, are provided in Table 5.

The results of the paired-*t* test show that the *p*-values was smaller then 0.05 for all algorithms. Accordingly, it can be concluded that the introduced HSA-SCA approach is significantly superior over all contenders for both thresholds alpha=0.1 and alpha=0.05.

## 5. Discussion

The average annual B[a]P concentrations of 3.73 $\mathrm{ng}\;m^{-3}$ and 2.78 $\mathrm{ng}\;m^{-3}$ in 2018 and 2019, respectively (Table 6), significantly exceeded the European Directive set level of 1 $\mathrm{ng}\;m^{-3}$. The maximum pollutant level reached 43.71 $\mathrm{ng}\;m^{-3}$ in the first year of the study period. At the same time, no values exceeded the critical threshold for the concentration of PM${}_{10}$, As, Cd, Ni, and Pb, and inorganic gaseous pollutants.

As indicated by the mean absolute SHAP values, the temperature at surface (TMPS), As, PM${}_{10}$, and total nitrogen oxide (NOx) concentrations appear to be major factors for governing B[a]P environmental fate (Table 7). In addition, the most important variables also include NO, SO${}_{2}$, Pb, and Cd concentrations, as well as the temperature at 2 m (T02M) and momentum flux intensity (MOFI)m have been shown to affect B[a]P dynamics. However, for this paper, we will focus on the aforementioned four.

### 5.1. Temperature at Surface

In this study, the temperature at the surface (TMPS) was estimated to be the most important parameter responsible for the B[a]P concentration increase of 1.17 $\mathrm{ng}\;m^{-3}$ on average, while mutual interrelations between TMPS and other studied parameters define three types of environmental conditions being responsible for shaping B[a]P levels. As a high-molecular-weight PAH, B[a]P is dominantly particle-bound in the atmosphere. The B[a]P partition between gas and particles is enhanced during colder months due to low temperature and high atmospheric pressure, which cause intense descending air movements and dry deposition of organic compounds [74]. Additionally, previous studies have shown that higher organic carbon content of particles in the cold season negatively affects the immobilization and biodegradation of PAHs [75], while high temperatures and light intensity in warm months enable both their photo- and biodegradation.

**Figure 7:**
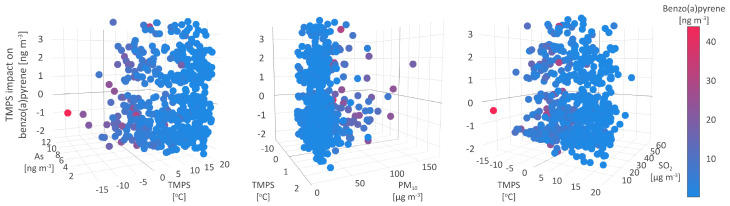
Temperature at surface impact on benzo(a)pyrene.

The first type of environment resulting in the increase of B[a]P concentrations up to 3.4 $\mathrm{ng}\;m^{-3}$ (Figure 7), was characterized by medium to low PM${}_{10}$, B[a]P, As, Cd, and Ni levels (35.2 $\mu g\;m^{-3}$, and 3.4, 1.4, 0.3, and 2.5 $\mathrm{ng}\;m^{-3}$ on average, respectively), medium to high NO and NOx concentrations (6.9 and 23.4 $\mu g\;m^{-3}$ on average, respectively), and meteorological parameters registered in a wide range of values. The observed constancy of the conditions suggests that this environment type might be related to anthropogenic sources, such as traffic and off-road vehicles.

In the second type of environment, TMPS was ambivalently related to the B[a]P concentrations, leading both to their decrease by up to −1 $\mathrm{ng}\;m^{-3}$ and the increase by up to 0.7 $\mathrm{ng}\;m^{-3}$. Compared to the first type, the second type of environment was characterized by lower B[a]P, As, Cd, Ni, Pb, PM${}_{10}$, NO${}_{2}$, and SO${}_{2}$ (2.0, 1.1, 0.2, 1.8, 3.8 $\mathrm{ng}\;m^{-3}$, and 32.8, 13.8, and 13.4 $\mu g\;m^{-3}$, respectively) and higher NO and NOx (about 9.5 and 28.8 $\mu g\;m^{-3}$, respectively) mean concentrations. The decrease in temperature range and wind speed (Figure 7) and the rise in relative humidity, alongside other meteorological parameters (MOFI, LIDS, and SHIF), indicate the stability of the atmosphere and cold weather-related conditions.

The third type of environment, leading to a decrease in B[a]P concentrations of 2.3 $\mathrm{ng}\;m^{-3}$, was associated with medium mean PM${}_{10}$, SO${}_{2}$, and As levels (34.6 and 14.1 $\mu g\;m^{-3}$, and 1.6 $\mathrm{ng}\;m^{-3}$, respectively), maximum observed B[a]P, As, and NO${}_{2}$ concentrations (43.7 and 13.6 $\mathrm{ng}\;m^{-3}$, and 98.5 $\mu g\;m^{-3}$, respectively), standard lifted index (304), and relative humidity (98%), minimum study period temperature (−15.3 ${}^{{}^{\circ}}$C), as well as the highest number of precipitation events, i.e., non-zero TPP6, CPP6, and CRAI values (Figure 7).

The atmospheric stability and the intensity of anthropogenic emissions during the cold part of the year seem to result in high B[a]P concentrations. Since PAHs are mostly particle-bound, and precipitation scavenging plays a significant role in the PM removal from the atmosphere, it could be expected that wet deposition represents a way of PM-bound B[a]P elimination from the atmosphere. As shown by Liu et al. [76], wet removal and photodegradation are up to 10 and 5 times, respectively, more efficient in B[a]P elimination during summer than in winter. Additionally, wet scavenging dominates as a B[a]P removal path in summer, while the impact of photodegradation outweighs the wet removal in winter.

### 5.2. Arsenic

This study suggests that As concentrations affect B[a]P level dynamics up to 0.9 $\mathrm{ng}\;m^{-3}$ on average (Table 7), more than any other pollutant. A few types of environment were distinguished by analyzing the interrelations between As and B[a]P and their coexistence within certain conditions.

The obtained interrelation indicates similar emission sources of inorganic As, in a mixture of arsenite (AsIII) and arsenate (AsV), and organic B[a]P in the air, that could be identified as high-temperature combustion of fossil fuels and wood [77,78]. In addition, because of low volatility, both As and B[a]P mostly exist as particle-bound in the atmosphere, particularly associated with fine aerosol fractions. Up to approximately 10% of B[a]P occurs in the gaseous phase [79], although the multiphase B[a]P distribution was also highly dependent on ambient temperature [80].

**Figure 8:**
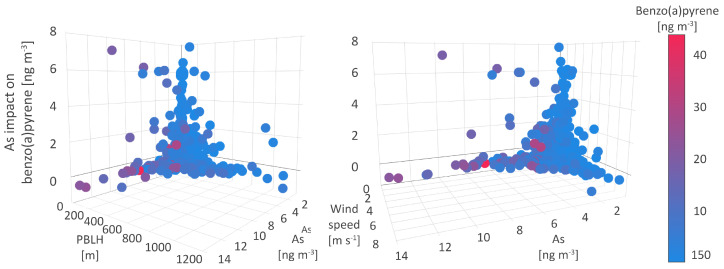
Arsenic impact on benzo(a)pyrene.

In the first type of environment, B[a]P concentrations exhibited an increase in the range from 4 to 7 $\mathrm{ng}\;m^{-3}$ (Figure 8), with maximum concentrations reaching 30 $\mathrm{ng}\;m^{-3}$. The relative impact of As, i.e., its association with B[a]P, compared to other studied parameters, reaches a maximum share of 43.6%. This environment was characterized by the lowest As and Cd concentrations, below 2 and 1.5 $\mathrm{ng}\;m^{-3}$, respectively, and low to medium NOx, SO${}_{2}$, and PM${}_{10}$ levels of below 55 $\mu g\;m^{-3}$, below 25 $\mu g\;m^{-3}$, and from 10 to 25 $\mu g\;m^{-3}$, respectively. Other PM-bound constituents, including Ni and Pb, were registered in higher concentrations of 8.2 and 24.6 $\mathrm{ng}\;m^{-3}$, respectively, which suggests the impact of local anthropogenic source emissions and dust resuspension, as well as the impact of occasional fossil fuel burning emissions. The co-occurrence of As and B[a]P was observed in the wide range of temperatures at the surface and 2 m (from 1 to 20 ${}^{{}^{\circ}}$C), which indicates that the relationship between As and B[a]P concentrations was not seasonally dependent. Additionally, this type of environment was featured by PBLH below 150 m, humidity above 74%, wind speed below 2 m s${}^{-1}$, and very low MOFI (Figure 8), all of which reflected extremely stable meteorological and atmospheric conditions, which were registered on a few occasions during the measurement campaign. Therefore, it can be assumed that in the first type of environment, the contributions of remote air pollution sources and atmospheric long-range transport to the observed B[a]P and As concentrations can be excluded.

The second type of environment was characterized by an increase in B[a]P concentrations up to 4 $\mathrm{ng}\;m^{-3}$ on average and by the lower impact of As (5 to 20%), relative to other pollutants. In comparison to the previous one, this environment was also marked by up to three times higher PM${}_{10}$ levels (70 $\mu g\;m^{-3}$), up to two times higher As (5 $\mathrm{ng}\;m^{-3}$) and NOx (up to 100 $\mu g\;m^{-3}$) levels, and somewhat higher SO${}_{2}$ (30 $\mu g\;m^{-3}$) concentrations. The assigned meteorological conditions included low humidity, air and soil temperatures ranging from −5 to 20 ${}^{{}^{\circ}}$C, PBLH below 480 m (Figure 8), and wind speed below 3.7 m s${}^{-1}$, as well as MOFI values typical for the cold season. As can be concluded, the second type of environment represented the cold season and its associated emissions of As and B[a]P as well as inorganic oxides from heating-related sources. In cold weather conditions, PM, NOx, SO${}_{2}$, and As are slow-reacting and the atmospheric reactions associated with the generation of secondary air pollutants (other oxide forms, sulfates, nitrates, or ozone), reaction byproducts or fine particles require a prolonged time, which in this case contributed to high pollutant concentrations assigned to the second type of B[a]P environment.

The third type of environment referring to the majority of measured pollutant concentrations recognized more than one pattern of As-B[a]P interrelations. Depending on the wind speed and other meteorological factors, both high and low B[a]P and As concentrations were registered. Namely, wind speed below 2 m s${}^{-1}$ was associated with the highest pollutant concentrations, while the increase in wind speed above 5 m s${}^{-1}$ resulted in a significant decrease in both pollutant concentrations below 1 $\mathrm{ng}\;m^{-3}$. These findings suggest a negligible contribution of regional pollutant sources to air quality at the sampling site, but also the presence of local pollution sources and processes, such as resuspension of ash from crude-oil and lignite-fired boilers, which strongly affect pollutant concentrations during the episodes of low wind speed.

SHAP values ranging from −0.6 to 0 $\mathrm{ng}\;m^{-3}$ referred to the situations in which As levels had a moderately negative or null impact on B[a]P dynamics. On these occasions, As, B[a]P, and PM${}_{10}$ levels were very high, 13.6 $\mathrm{ng}\;m^{-3}$, 22 $\mathrm{ng}\;m^{-3}$ and 177 $\mu g\;m^{-3}$, respectively, while the SO${}_{2}$ and NOx levels did not exceed 10 $\mu g\;m^{-3}$. Given these findings were associated with the T02M range from −3 to 5 ${}^{{}^{\circ}}$C, we can assume that As and B[a]P have separate sources during the cold season, which contribute to high concentrations of either one or another pollutant. More data and further analysis could provide detailed insight and confirm these assumptions.

### 5.3. Particulate Matter

The PM${}_{10}$ concentration is the third significant parameter that affects B[a]P concentrations, as shown by the mean absolute SHAP value of 0.8 $\mathrm{ng}\;m^{-3}$. In the absence of meteorological conditions favoring the association of B[a]P and small particle fraction, the relationship between PM${}_{10}$ and B[a]P stands out.

**Figure 9:**
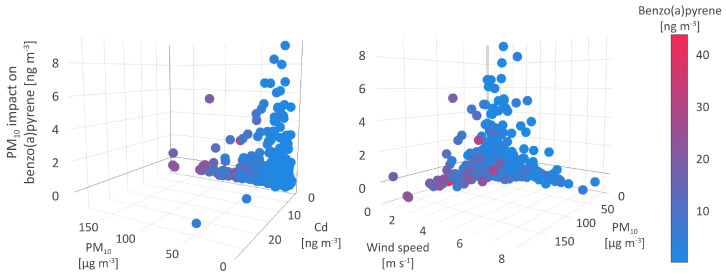
Particulate matter impact on benzo(a)pyrene.

The highest observed positive associations between PM${}_{10}$ levels and B[a]P concentrations, in compliance with a relative share of 57.52% and assigned an absolute SHAP value of 8.36 $\mathrm{ng}\;m^{-3}$, was registered in the environmental conditions associated with the lowest concentrations of all pollutants, including PM${}_{10}$ levels below 32 $\mu g\;m^{-3}$. As regards meteorological conditions, the strongest interrelation between PM${}_{10}$ and B[a]P concentrations was detected in the environment characterized by air and soil temperatures ranging from 0 to 20 ${}^{{}^{\circ}}$C and low wind speed (below 2 m s${}^{-1}$). This type of environment is not seasonally specific and might indicate natural interactions in the atmosphere such as associations between PAHs and PM. Atmospheric PAHs such as high-ring B[a]P are easily adsorbed onto suspended particles with high organic content [76] while the degradation of particle-bound B[a]P fraction is minimized or inhibited. The gas-to-particle partitioning of pollutants and atmospheric removal by wet scavenging are favored depending on the atmospheric conditions, PM surface, its composition and size, and contaminant properties [81]. In the warm season, the increase in temperatures leads to increased B[a]P volatility, followed by its biodegradation. As the impact of PM${}_{10}$ on B[a]P levels weaken the environmental conditions change slightly towards higher pollutant concentrations and an increase in wind speed and PBLH (Figure 9).

Given the SHAP value of −0.87 PM${}_{10}$, a high number of registered medium to high B[a]P concentrations was negatively associated with PM${}_{10}$, particularly in the environment of high suspended particles As and low Cd, Ni, Pb, NOx, and SO${}_{2}$ levels. As regards meteorological conditions, these interactions took place during the coldest days of the winter period, when low PBLH, high cloudiness, and wind speed up to 3 m s${}^{-1}$ were recorded (Figure 9).

As previously mentioned, the cold season was the period of intense emissions from power plants, domestic heating units, and commercial sources, resulting in elevated levels of PM, especially those of smaller diameter (PM${}_{2.5}$ and PM${}_{1}$) rather than PM${}_{10}$. The finest particle fractions represented a highly suitable matrix for the adsorption of PAHs and these associations could be a possible explanation for the negative relation between B[a]P and PM. A number of studies have shown that small particle diameter plays an important role in the entrapment of PAHs, and thus more than 70% of high-molecular-weight PAHs with higher octanol-water partition coefficients, including B[a]P, is PM${}_{2.5}$-bound [82,83]. Low air temperature, wind speed, solar radiation, and PBLH inhibited the vertical diffusion of pollutants and enhanced gas-to-particle pollutant partitioning [84,85]. In addition to this, the strong adsorption capacity of fine PM fraction prevailed over other environmental factors and suggests the particle partition of B[a]P to PM${}_{2.5}$ and a smaller fraction rather than to PM${}_{10}$. Lobscheid et al. [86] used multivariate linear regression models to predict relations of ambient B[a]P levels and PM${}_{2.5}$ concentrations, spatial, temporal, and meteorological variates. The most significant variables included the average daily PM${}_{2.5}$ concentration, wind speed, temperature, and relative humidity.

In contrast to this, during the warm and windy season, when the average temperatures, wind speed, and PBLH exceeded 15 ${}^{{}^{\circ}}$C, 4 m s${}^{-1}$, and 450 m, respectively, the concentrations of PM${}_{10}$ and their constituents exhibited a significant decrease, although the same does not apply for NOx and SO${}_{2}$. High solar radiation and temperature in warmer seasons lead to the dispersion and photochemical degradation of the majority air polluting species [80,87], but the persistence of medium to high gaseous oxide levels during the warm season indicated the impact of intense and year-round continuous traffic emissions at the sampling site.

### 5.4. Nitrogen Oxides

Similar to PAHs, NOx (NO and smaller share of NO${}_{2}$) emissions mainly resulted from the high-temperature combustion processes in power plants and motor vehicles. Both groups of compounds, PAHs and NOx, were subject to photochemical reactions in the atmosphere. Besides undergoing gas-particle phase distribution, PAHs are precursors for the generation of nitro-compounds. Namely, in the presence of free radicals, OH-PAH or NO${}_{3}$-PAH are formed and subsequently, in the few-hour reaction with NO${}_{2}$ upon release of nitric acid or water molecule, nitro-PAHs were generated [88,89].

**Figure 10:**
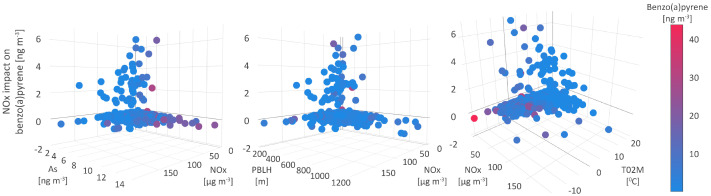
Nitrogen oxides impact on benzo(a)pyrene.

The mean absolute SHAP value of 0.6 $\mathrm{ng}\;m^{-3}$ defines NOx as the third most significant parameter for shaping B[a]P levels in two distinguished types of environment, one of which strongly supports the increase in B[a]P concentrations. The polluted environment, with moderate to high B[a]P levels (average value of 3 $\mathrm{ng}\;m^{-3}$) and attributed SHAP value of 6.78 $\mathrm{ng}\;m^{-3}$, was characterized by a wide range of NOx, PM${}_{10}$, and SO${}_{2}$ concentrations, from 1.28 to 144 $\mu g\;m^{-3}$, up to 70 $\mu g\;m^{-3}$ and up to 30 $\mu g\;m^{-3}$, respectively; however, the lowest levels of PM-bound As, Cd, Ni, and Pb (Figure 10).

The meteorological conditions which enabled the positive impact of NOx on modelled B[a]P level dynamics and a wide range of B[a]P, NOx, PM${}_{10}$, and SO${}_{2}$ concentrations, refer to stable high-humidity cold weather without precipitations, with wind speed and PBLH below 3 m s${}^{-1}$ and 400 m, respectively; temperatures in the range from −7 to 20 ${}^{{}^{\circ}}$C, as well as with the corresponding CAPE, CPP6, CRAI, MOFI, and SHIF values (Figure 10). Under these conditions, common emission sources (fossil fuel burning for heating purposes) of the listed pollutants were intensified leading to their higher concentrations. In addition, the stagnant high-humidity conditions during heavy haze events enhanced the transformation of primary emitted particles containing PAHs to secondary organic aerosol (SOA), with the prominent presence of sulfate and nitrate water-soluble species dissolved in an aqueous outer particle layer [90].

The majority of studied pollutant events can be distinguished into two groups depending on the SHAP values and the strength of NOxs negative impact on modeled B[a]P concentration dynamics. The type of environment in which NOx and B[a]P interrelations are expressed by a lower SHAP value of −1.76 $\mathrm{ng}\;m^{-3}$, refers to the warm season, with air temperatures from 15 to 20 ${}^{{}^{\circ}}$C, an occasional wind of high speed from 5 to 8 m s${}^{-1}$ and mean daily PBLH above 1000 m. As can be expected, these meteorological conditions have favored pollutant dispersion and resulted in low pollutant concentrations, as confirmed by measurements (Figure 10). During the warm season, PAHs undergo photolysis or processes which can yield their derivative compounds, such as oxygenated and nitrated PAHs. The UV-mediated ozone photolysis is a source of OH radicals in the troposphere, which react with PAHs to produce intermediate compounds OH-PAHs. After substitution with NO${}_{2}$, OH-PAHs are further converted to nitro-PAHs, particularly at night, when the concentrations of NO are low [88]. Additionally, nitro-PAHs are also generated in the chemical reactions between PAHs and NO${}_{3}$-radicals, originating from reactions between O${}_{3}$ and NO, and their formation can explain the negative NOx and B[a]P interrelations.

The SHAP value of −0.3 $\mathrm{ng}\;m^{-3}$ was attributed to the environment where no significant interactions between NOx and B[a]P were registered. Occasionally, these events were characterized either by high concentrations of B[a]P, PM${}_{10}$, PM-bound constituents, and low NOx levels, or the opposite, the lowest concentrations of suspended particles, their constituents and high NOx levels exceeding 50 $\mu g\;m^{-3}$, which implies two different sources of origin.

## 6. Conclusions

In this study, we employed, coupled, and optimized advanced artificial intelligence-based modeling to accurately interrelate air pollution-related parameters to capture defining factors and processes that shape benzo(a)pyrene behavior. We have applied the XGBoost model optimized by metaheuristics and the Shapley Additive exPlanations explainable artificial intelligence method to a two-year database of pollutant concentrations and meteorological parameters to characterize types of environments that govern the interactions between benzo(a)pyrene, other polluting species, and meteorological conditions. The results suggest that the hybrid self-adaptive sine cosine algorithm method displayed a supreme performance level, by achieving the best scores for key performance indicators (mean square error of 2.5 and $R^{2}$ of 0.9), while the firefly algorithm scored the best results for standard deviation and variance, by delivering the most stable results. As shown, the temperature at the surface, arsenic, PM${}_{10}$, and NOx were recognized to affect 22.7%, 14.4%, and 10.0% of benzo(a)pyrene concentrations, respectively. The observed interrelation between particulates and inorganic and organic pollutants could be associated with intensified fossil fuel burning such as low-quality lignite coal during the cold season. In the conditions of low temperature, PM, NOx, SO${}_{2}$, and As are slow-reacting, and the atmospheric reactions in which the pollutants are involved require a prolonged time, which in this case enhanced the pollutant ambient levels. In addition, during cold seasons, photodegradation of B[a]P was weakened and its adsorption to the particles was favored. The results of this study have proved the potential of the applied methodology to improve the scientific knowledge and understanding of the complex factors that govern the environmental fate of air-polluting species.

## Figures and Tables

**Table 1:** Comparative results of the objective function (MSE) of the observed metaheuristics.

Method	HSA-SCA	SCA	GA	PSO	ABC	FA	WOA	HHO	ChOA
Best	**2.468293**	3.184137	3.192827	3.077008	3.153481	2.590850	3.206221	3.180909	3.129932
Worst	**2.893362**	3.605363	3.635218	3.773639	3.774347	2.918338	3.685749	3.584440	3.561904
Mean	**2.731538**	3.443475	3.413906	3.390799	3.369339	2.771516	3.466122	3.363900	3.379943
Median	**2.725915**	3.479927	3.423980	3.413375	3.306318	2.779478	3.472304	3.358337	3.371960
Std	0.114964	0.112574	0.144976	0.214997	0.157270	**0.097592**	0.109602	0.130492	0.112995
Var	0.013217	0.012673	0.021018	0.046224	0.024734	**0.009524**	0.012013	0.017028	0.012768

**Table 2:** Detailed metrics for the best individual run of the observed metaheuristics.

	$R^{2}$	R	MAE	MSE	RMSE	IA
HSA-SCA	**0.892845**	**0.944905**	0.987179	**2.468293**	**1.571080**	**0.970348**
SCA	0.861769	0.928315	1.081976	3.184137	1.784415	0.960579
GA	0.861392	0.928112	1.056114	3.192827	1.786848	0.958925
PSO	0.866420	0.930817	1.096070	3.077008	1.754140	0.959703
ABC	0.863100	0.929032	1.085311	3.153481	1.775804	0.959175
FA	0.887525	0.942085	**0.981363**	2.590850	1.609612	0.967438
WOA	0.860810	0.927799	1.036537	3.206221	1.790592	0.961817
HHO	0.861909	0.928391	1.143855	3.180909	1.783510	0.961245
ChOA	0.864122	0.929582	1.048337	3.129932	1.769161	0.960678

**Table 3:** Best solutions’ determined XGBoost hyper-parameters set.

Method	l.r. (μ)	Max_child_weight	Subsample	Collsample_bytree	Max_depth	Gamma
HSA-SCA	0.535844	4.768378	0.920331	0.899994	5	0.037125
SCA	0.424673	6.830426	0.903051	1.000000	10	0.800000
GA	0.515505	1.239850	0.921408	1.000000	4	0.000000
PSO	0.469675	5.890036	0.966332	0.732996	7	0.514569
ABC	0.424717	6.756257	0.910125	0.797826	7	0.349785
FA	0.473028	5.459757	0.937122	1.000000	7	0.318114
WOA	0.518772	6.961853	0.976281	0.978017	4	0.408959
HHO	0.533272	6.254540	1.000000	1.000000	10	0.800000
ChOA	0.388340	2.995555	0.766726	1.000000	8	0.000000

**Table 4:** Shapiro-Wilk scores for validating the normality condition.

Methods	HSA-SCA	SCA	GA	PSO	ABC	FA	WOA	HHO	ChOA
	0.362	0.351	0.198	0.145	0.312	0.304	0.342	0.263	0.347

**Table 5:** Shapiro-Wilk scores over the mean differences between two samples as prerequisite for paired-*t* test, accompanied by the paired-*t* test results.

Methods vs. HSA-SCA	SCA	GA	PSO	ABC	FA	WOA	HHO	ChOA
**Shapiro-Wilk**	0.164	0.202	0.241	0.195	0.213	0.224	0.189	0.207
**paired-*****t*** **test**	0.021	0.024	0.025	0.031	0.041	0.025	0.031	0.033

**Table 6:** Descriptive statistics.

Year	Statistics	B[a]P	PM${}_{10}$	As	Cd	Ni	Pb	SO${}_{2}$	NO	NO${}_{2}$	NOx
		[$\mathrm{ng}\;m^{-3}$]	[$\mu g\;m^{-3}$]	[$\mathrm{ng}\;m^{-3}$]	[$\mathrm{ng}\;m^{-3}$]	[$\mathrm{ng}\;m^{-3}$]	[$\mathrm{ng}\;m^{-3}$]	[$\mu g\;m^{-3}$]	[$\mu g\;m^{-3}$]	[$\mu g\;m^{-3}$]	[$\mu g\;m^{-3}$]
2018	Average	3.73	36.18	1.56	0.41	2.78	4.35	10.82	10.63	17.88	29.96
	Minimum	0.07	10.2	0.5	0.05	1.5	2.5	2.09	0.5	0.5	0.5
	Maximum	43.71	179.7	11.2	27.1	19.6	30.7	25.02	101.49	98.5	189.71
	Median	1.15	29	0.5	0.2	1.5	2.5	12.45	4.61	14.33	19.3
2019	Average	2.78	33.01	1.38	0.2	2.26	4.26	17.21	4.98	11.91	19.43
	Minimum	0.03	4.9	0.5	0.05	1.5	2.5	5.7	1.1	1.75	6.1
	Maximum	23.63	180.7	13.6	1.9	28.5	40.6	66.3	91.2	48	146.81
	Median	1.07	23.3	0.5	0.1	1.5	2.5	15.45	2.75	10.45	14.6

**Table 7:** SHAP values.

Parameter	TMPS	As	PM${}_{10}$	NOx	NO	SO${}_{2}$	TO2M	Pb	MOFI	LIB4	SHIF	LHTF
Absolute	1.17	0.906	0.796	0.608	0.321	0.247	0.192	0.161	0.158	0.158	0.141	0.131
Relative [%]	22.7	14.36	13.75	9.99	4.99	4.25	3.98	3.38	2.45	2.99	2.18	2.33

## Abbreviations

The following abbreviations are used in this manuscript:

SHAP	SHapley Additive exPlanations	
XGBoost	eXtreme Gradient Boosting	
**Label**	**Meteorological paramater**	**Unit**
PRSS	Pressure at surface	hPa
MSLP	Pressure reduced to mean sea level	hPa
TPP6	Accumulated precipitation (6 h accumulation)	m
MOFI	Momentum flux intensity (3- or 6-h average)	$N\;m^{-2}$
MOFD	Momentum flux direction (3- or 6-h average)	${}^{{}^{\circ}}$
SHTF	Sensible heat net flux at surface (3- or 6-h average)	$W\;m^{-2}$
DSWF	Downward short wave radiation flux (3- or 6-h average)	$W\;m^{-2}$
RH2M	Relative Humidity at 2 m AGL	%
WS	Wind speed at 10 m AGL	$m\;s^{-1}$
WD	Wind direction at 10 m AGL	${}^{{}^{\circ}}$
TO2M	Temperature at 2 m AGL	${}^{{}^{\circ}}$C
TCLD	Total cloud cover (3- or 6-h average)	%
SHGT	Geopotential height	gpm *
CAPE	Convective available potential energy	$J\;{\mathrm{Kg}}^{-1}$
CINH	Convective inhibition	$J\;{\mathrm{Kg}}^{-1}$
LISD	Standard lifted index	${}^{{}^{\circ}}$C
LIB4	Best 4-layer lifted index	${}^{{}^{\circ}}$C
PBLH	Planetary boundary layer height	m
TMPS	Temperature at surface	${}^{{}^{\circ}}$C
CPP6 **	Accumulated convective precipitation (6 h accumulation)	m
SOLM	Volumetric soil moisture content	frac.
CSNO	Categorial snow (yes = 1, no = 0) (3- or 6-h average)	
CICE	Categorial ice (yes = 1, no = 0) (3- or 6-h average)	
CFZR	Categorial freezing rain (yes = 1, no = 0) (3- or 6-h average)	
CRAI	Categorial rain (yes = 1, no = 0) (3- or 6-h average)	
LHTF	Latent heat net flux at surface (3- or 6-h average)	W/m${}^{2}$
LCLD	Low cloud cover (3- or 6-h average)	%
MCLD	Middle cloud cover (3- or 6-h average)	%
HCLD	High cloud cover (3- or 6-h average)	%
* geopotential meters		
** Beginning with 00 UTC July 15, 2019, CPPA (total accumulation) instead of CPP6 (6-h accumulation)		
